# Norepinephrine weaning in septic shock patients by closed loop control based on fuzzy logic

**DOI:** 10.1186/cc7149

**Published:** 2008-12-09

**Authors:** Mehdi Merouani, Bruno Guignard, François Vincent, Stephen W Borron, Philippe Karoubi, Jean-Philippe Fosse, Yves Cohen, Christophe Clec'h, Eric Vicaut, Carole Marbeuf-Gueye, Frederic Lapostolle, Frederic Adnet

**Affiliations:** 1 Samu 93 – EA 3409, Université Paris 13, Hôpital Avicenne, Rue de Stalingrad, 93000 Bobigny, France; 2 Département d'Anesthésie et de Réanimation, Hôpital Ambroise Paré, Avenue Charles-de-Gaulle, 92100 Boulogne Billancourt, France; 3 Service de Réanimation, Hôpital Avicenne, Rue de Stalingrad, 93000 Bobigny, France; 4 Department of Surgery (Emergency Medicine), University of Texas Health Science Center at San Antonio, Medical Drive, San Antonio, TX 78229, USA; 5 Unité de Recherche Clinique, Hôpital Fernand Widal, Rue Ambroise Paré, 75475 Paris Cedex, France; 6 BioMoCeTi, UMR 7033, UFR SMBH, Université Paris 13, Rue Marcel Cachin, 93000 Bobigny, France

## Abstract

**Introduction:**

The rate of weaning of vasopressors drugs is usually an empirical choice made by the treating in critically ill patients. We applied fuzzy logic principles to modify intravenous norepinephrine (noradrenaline) infusion rates during norepinephrine infusion in septic patients in order to reduce the duration of shock.

**Methods:**

Septic patients were randomly assigned to norepinephrine infused either at the clinician's discretion (control group) or under closed-loop control based on fuzzy logic (fuzzy group). The infusion rate changed automatically after analysis of mean arterial pressure in the fuzzy group. The primary end-point was time to cessation of norepinephrine. The secondary end-points were 28-day survival, total amount of norepinephine infused and duration of mechanical ventilation.

**Results:**

Nineteen patients were randomly assigned to fuzzy group and 20 to control group. Weaning of norepinephrine was achieved in 18 of the 20 control patients and in all 19 fuzzy group patients. Median (interquartile range) duration of shock was significantly shorter in the fuzzy group than in the control group (28.5 [20.5 to 42] hours versus 57.5 [43.7 to 117.5] hours; *P *< 0.0001). There was no significant difference in duration of mechanical ventilation or survival at 28 days between the two groups. The median (interquartile range) total amount of norepinephrine infused during shock was significantly lower in the fuzzy group than in the control group (0.6 [0.2 to 1.0] μg/kg versus 1.4 [0.6 to 2.7] μg/kg; *P *< 0.01).

**Conclusions:**

Our study has shown a reduction in norepinephrine weaning duration in septic patients enrolled in the fuzzy group. We attribute this reduction to fuzzy control of norepinephrine infusion.

**Trial registration:**

Trial registration: Clinicaltrials.gov NCT00763906.

## Introduction

Despite advances in critical care, the death rate from severe sepsis remains approximately 30% to 50%. In 1995, severe sepsis accounted for 9.3% of all deaths in the USA [1]. It is generally agreed that fluid resuscitation and vasopressors should be initiated promptly to treat shock and organ failure, and rapidly restore the mean arterial pressure (MAP) to 60 to 90 mmHg [2,3].

The vasopressor in most common use is norepinephrine (noradrenaline) but, because of its weak inotropic effect and concerns about regional blood flow, dobutamine is often administered concomitantly. As soon as haemodynamic variables are stable, vasopressor and inotropic support is gradually weaned in order to decrease duration of shock and avoid adrenoreceptor downregulation and catecholamine refractoriness [4]. However, there is little published evidence on how to wean support. The weaning rate is usually chosen empirically because conventional quantitative models cannot cope with the complexity of the biological systems involved.

Closed-loop control based on fuzzy logic permits the use of conventional symbolic systems (specified in the form of tabulated rules) in continuous form and can ensure stability through adaptive self-organizing control. It has been applied to supervisory control in several medical fields [5]. For instance, a multiple drug haemodynamic supervisory control system has been developed for controlling MAP and cardiac output [6]. However, to our knowledge, there are very few randomized controlled trials comparing fuzzy logic decisions with human decisions by practitioners [7,8].

We compared, in a prospective, randomized pilot study, the duration of weaning of norepinephrine as determined by a closed-loop control based on fuzzy logic algorithm versus manual control by the clinician in patients with septic shock. Our goal was to reduce the duration of poorly controlled haemodynamic status by using a closed-loop controller based on fuzzy logic in septic patients.

## Materials and methods

### Approval of study design and informed consent

This prospective, randomized controlled trial was conducted in the 16-bed intensive care unit (ICU) of Avicenne University Hospital. The study was approved by the Consultative Council for the Protection of Persons Volunteering for Biomedical Research of Aulnay Hospital. Throughout the study the clinical coordinating center (Association pour le Développement de la Recherche et l'Enseignement de la Médecine d'Urgence (ADREMU), Bobigny) was available 24 hours a day to answer investigators' questions about patient eligibility and safety, and to deal with any reported serious adverse events.

Requirement for informed consent was waived because patients were under mechanical ventilation and sedated. However, written informed consent was obtained from patients' authorized representatives upon entry into the study and from the patients themselves for use of their individual data, as soon as their clinical status made this possible.

### Eligibility

Patients were enrolled consecutively from December 2004 through January 2006 and were eligible for entry into the study if they had known or suspected infection according to clinical criteria and if, within the previous 24 hours, they had manifested three or more signs of a systemic inflammatory response syndrome and sepsis-induced dysfunction of at least one organ or system that lasted for less than 24 hours. The criteria for severe sepsis were those defined by Bernard and coworkers [9]. In addition, for inclusion, norepinephrine infusion had to be begun within 24 hours before randomization and had to have been in use for at least 6 hours but for less than 24 hours. Exclusion criteria were age less than 18 years, pregnancy, weight above 135 kg, requirement for continuous epinephrine infusion, severe head injury, stroke and comatose state after cardiac arrest.

Baseline characteristics including demographics, history and type of infection, and laboratory test results were obtained within the 24 hours before randomization. Disease severity at baseline was assessed using the Simplified Acute Physiology score II and the Sequential Organ Failure Assessment score [10,11].

### Treatment

Patients were randomly assigned to norepinephrine infused either at the clinician's discretion or under closed-loop control based on fuzzy logic. The method of randomization was a one-to-one allocation. The target MAP was 65 to 75 mmHg (depending on the underlying condition of the patient), measured using a Siemens SC9000 monitor (Siemens, Amsterdam, The Netherlands).

**Figure 1:**
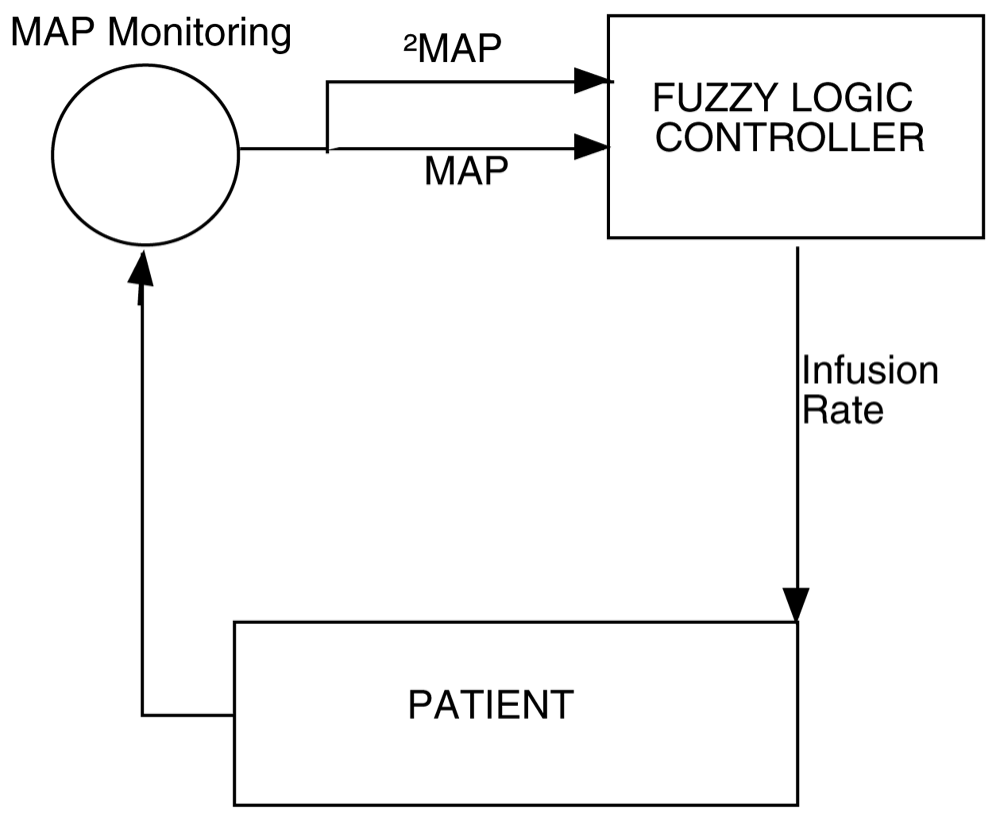
Scheme for a fuzzy logic based norepinephrine controller. The monitor was connected to a computer that converted the mean arterial pressure (MAP) and norepinephrine infusion rate into fuzzy datasets and automatically calculated the required change in rate of infusion. MAP level and MAP variation (ΔMAP) – the variables to be controlled – are the outputs of the controlled system, whereas the norepinephrine infusion rate is the input to be adjusted to reach the desired MAP value. The infusion rate changed automatically every 7 minutes after analysis of the MAP and the ΔMAP.

In the control group, the intensivist adapted norepinephrine doses to the haemodynamic status of the patient. There was no nurse norepinephrine weaning protocol in our ICU. In the 'fuzzy' group patients, the monitor was connected to a computer that converted the MAP and norepinephrine infusion rate into fuzzy datasets and automatically calculated the required change in rate of infusion. MAP control can be viewed in terms of engineering control theory (Figure 1). MAP level and MAP variation (ΔMAP) – the variables to be controlled – are the inputs of the controlled system, whereas the norepinephrine infusion rate is the output to be adjusted to achieve the desired MAP value. The computer was in turn connected to an automated syringe pump (Fresenius Vial Inc., Brezins, France). Fuzzy set theory is summarized in the additional materials [see Additional data file 1]. The infusion rate changed automatically every 7 minutes after analysis of the MAP and the ΔMAP. It could change by +1 to +20% (or -1% to -20%) without human oversight. However, all changes in rate greater than 40% (two consecutive changes of 20%) when the infusion rate of norepinephrine was superior to 1 mg/hour had to be validated by the clinician. When it occurred, an audible alarm sounded and a member of the medical team was required to validate (or not) the change proposed by the computer after patient assessment. At any time, the intensivist could interrupt the computer control and change the dosage manually if the patient's condition required it. A study manager (FA or MM) was available 24 hours per day and 7 days per week while any patient was included in the study to give advice if an abnormality occurred. Other safety control included the sounding of an alarm if the computer was disconnected.

In order to obtain an accurate MAP measurement with the least possible number of artefacts (caused by flushes, bends, knotting or blood sampling on the arterial lines), we measured MAP every 10 seconds for 7 minutes and then processed all obtained values with median values filtering. Moreover, this filter eliminated all artefact-predefined values. Thus, the MAP regulated by the algorithm is calculated from a set of 42 values of MAP. The reason for applying such a process to the data obtained from arterial measures was to increase their resistance to artefacts.

In order to avoid any modification concerning management of enrolled patients, all intensivists were blinded to the end-point definitions of our study (duration of norepinephrine weaning, mortality and duration of mechanical ventilation). Only two independent researchers (MM and FA) were permitted to perform adjustments to the computer. These investigators were not directly involved in patient care.

### Outcome measures

Upon inclusion, patients were followed throughout their ICU stay after or until death. The following were recorded daily while the patients were receiving norepinephrine in the ICU: vital signs, standard laboratory variables, results for cultures of specimens from new infection sites, and interventions. The primary end-point was duration of weaning, defined as cessation of vasopressor support [12]. Vasopressor support was defined as a norepinephrine dose above 0.1 mg/hour. The secondary end-points were 28-day survival, total amount of norepinephine infused, duration of mechanical ventilation and length of stay in the ICU.

### Statistical analysis

We hypothesized that closed-loop control of infusion would reduce norepinephrine weaning time by 45%. We calculated that we would require 20 patients per group to detect a 45% reduction, assuming a 5% α error, a 10% β error and 90% power. We used Kaplan-Meier curves to analyze probable duration of vasopressor treatment, mechanical ventilation and survival. To compare the two groups, we used the log-rank test or competitive risk analysis when the occurrence of death could interact with the event under study (for instance, the event 'cessation of mechanical ventilation' may occur because the patient no longer requires mechanical ventilation or because he or she died) [13]. All of the tests were two-sided at 5% significance levels. All calculations were made using SAS 9.1.3 (SAS Institute, Cary, NC, USA).

## Results

### Baseline characteristics

We evaluated 42 patients. Three patients were removed from the study; two patients were excluded because they did not meet the eligibility criteria and one patient was excluded because of a technical problem. The remaining 39 patients were randomly assigned either to the fuzzy group (n = 19) or to the control group (n = 20).

Demographics, disease severity, haemodynamic variables, and the type and anatomical site of the underlying infection were similar in the control and fuzzy groups (Table 1).

**Table 1:** Baseline characteristics of the patients

Variable	Control group (n = 20)	Fuzzy group (n = 19)
Age (years)	66 ± 12	64 ± 12
Male sex	70%	58%
Weight (kg)	67 ± 15	71 ± 14
Prior or coexisting conditions		
Chronic obstructive pulmonary disease	40%	37%
Congestive cardiomyopathy	0%	5%
Diabetes	10%	5%
Liver disease	2%	0%
Chronic renal failure	0%	0%
Cancer	30%	26%
Activity limitation (A/B/C/D)^a^	3/8/7/2	4/10/5/0
McCabe classification (1/2/3)^b^	10/6/4	12/4/3
Recent surgical history	20%	16%
Disease severity		
Temperature (°C)	36.8 ± 1.8	37.7 ± 1.5
Heart rate (beats/minute)	119 ± 26	103 ± 27
Systolic blood pressure (mmHg)	84 ± 14	80 ± 22
Mean blood pressure (mmHg)	61 ± 12	59 ± 16
White cell count (/mm^3^)	16,100 ± 8,800	17,600 ± 12,000
Platelet (× 10^3^/μl)	263 ± 198	262 ± 138
Haematocrit (%)	30 ± 7	32 ± 6
Blood urea nitrogen (mmol/l)	10.3 ± 6.3	10.4 ± 6.2
Creatinine (μmol/l)	113 ± 70	136 ± 88
Total bilirubin (μmol/l)	12.3	15.5
Lactate (mmol/l)	2.5 ± 2.1	2.0 ± 0.8
PaO_2_/FiO_2 _(mmHg)	166 ± 81	181 ± 93
Arterial pH	7.28 ± 0.14	7.31 ± 0.09
SAPS II scale	62 ± 23	58 ± 16
Initial SOFA score	11.0 ± 2.5	11.2 ± 3.3
Norepinephrine infusion at the time of randomization (μg/kg per minute)	0.6 ± 0.4	0.8 ± 0.4

Site of infection^c^		
Lung	75%	63%
Abdomen	25%	10%
Urinary tract	5%	15%
Other	5%	10%
Bacterial pathogen staining		
Gram-negative	40%	58%
Gram-positive	25%	10%
Unconfirmed	35%	32%

Values are expressed as number, percentage or mean ± standard deviation. Percentages may not total 100 because of rounding. ^a^Levels of activity were defined as follows (Knaus Chronic Health Status score): A = prior good health, no functional limitations; B = mild to moderate limitation of activity because of a chronic medical problem; C = chronic disease producing serious but not incapacitating limitation of activity; and D = severe restriction of activity due to disease, includes persons bedridden or institutionalized due to illness. ^b^McCabe classification: 1 = nonfatal disease; 2 = ultimately fatal disease; and 3 = rapidly fatal disease. ^c^The site of infection was either documented or presumed on the basis of clinical findings. PaO_2_/FiO_2_, arterial oxygen tension/fractional inspuired oxygen ratio; SAPS, Simplified Acute Physiology Score; SOFA, Sequential Organ Failure Assessment.

Four patients received dobutamine (no significant difference between the two groups) and one patient received epinephrine after randomization. There was no difference in crystalloid/colloid infusion volumes during the period of shock between the two groups.

### End-points

**Figure 2:**
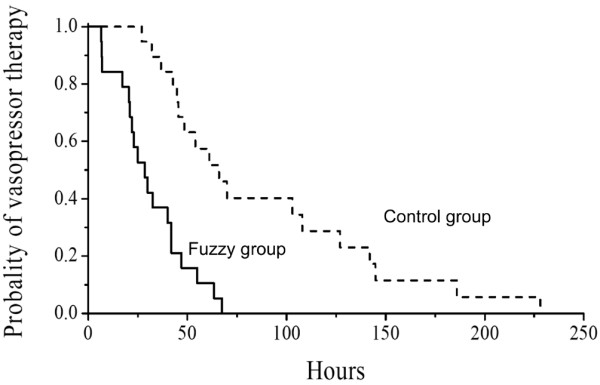
Kaplan-Meier curves demonstrating the probability of being on norepinephrine therapy during the study. Comparisons between the time distribution of both groups were performed by means of the generalized Wicolxon (Breslow) test. Competitive risk analysis was performed when the occurrence of death interacted with the event under study (two patients); *P *< 0.0001.

Weaning of norepinephrine was achieved in 18 of the 20 control patients and in all 19 fuzzy group patients. Duration of shock was significantly shorter (*P *< 0.001) in the fuzzy group than in the control group (Figure 2). The median time of vasopressor support was 28.5 hours (interquartile range = 20.5 to 42 hours) in the fuzzy group and 57.5 hours (interquartile range = 43.7 to 117.5 hours) in the control group. Two control group patients (5.1%) died before norepinephrine weaning was completed (no significant difference between groups), corresponding to the weaning failures. There was no significant difference in duration of mechanical ventilation between the two groups (Table 2). The total amount of norepinephrine infused was significantly lower in the fuzzy group than in the control group (Table 2).

**Table 2:** Outcome measures

Measure	Control group (n = 20)	Fuzzy group (n = 19)	*P*
Shock duration (hours)	57.5 (43.7–117.5)	28.5 (20.5–42)	<0.0001
Weaning failure (%)	2 (10)	0 (0)	0.49
Mortality at 28 days (%)	7 (37)	7 (35)	0.80
Mechanical ventilation (days)	20 (11–32)	15 (7–38)	0.96
ICU-free days at day 28	7 (0–18)	3 (0–17)	0.74
Total norepinephrine infused^a ^(μg/kg)	1.4 (0.6–2.7)	0.6 (0.2–1.0)	<0.01
Crystalloid/colloid^a ^(ml)	8750 (6000–14000)	6000 (3275–7512)	0.47

Values are expressed as median (interquartile range). ^a^Total amount administered during shock. ICU, intensive care unit.

Twenty-eight days after inclusion, seven out of 19 patients in the fuzzy group (37%) and seven out of 20 (35%) of the patients in the control group had died. The difference between groups in the rate of death from any cause was not significant. A Kaplan-Meier analysis of survival yielded similar results.

**Figure 3:**
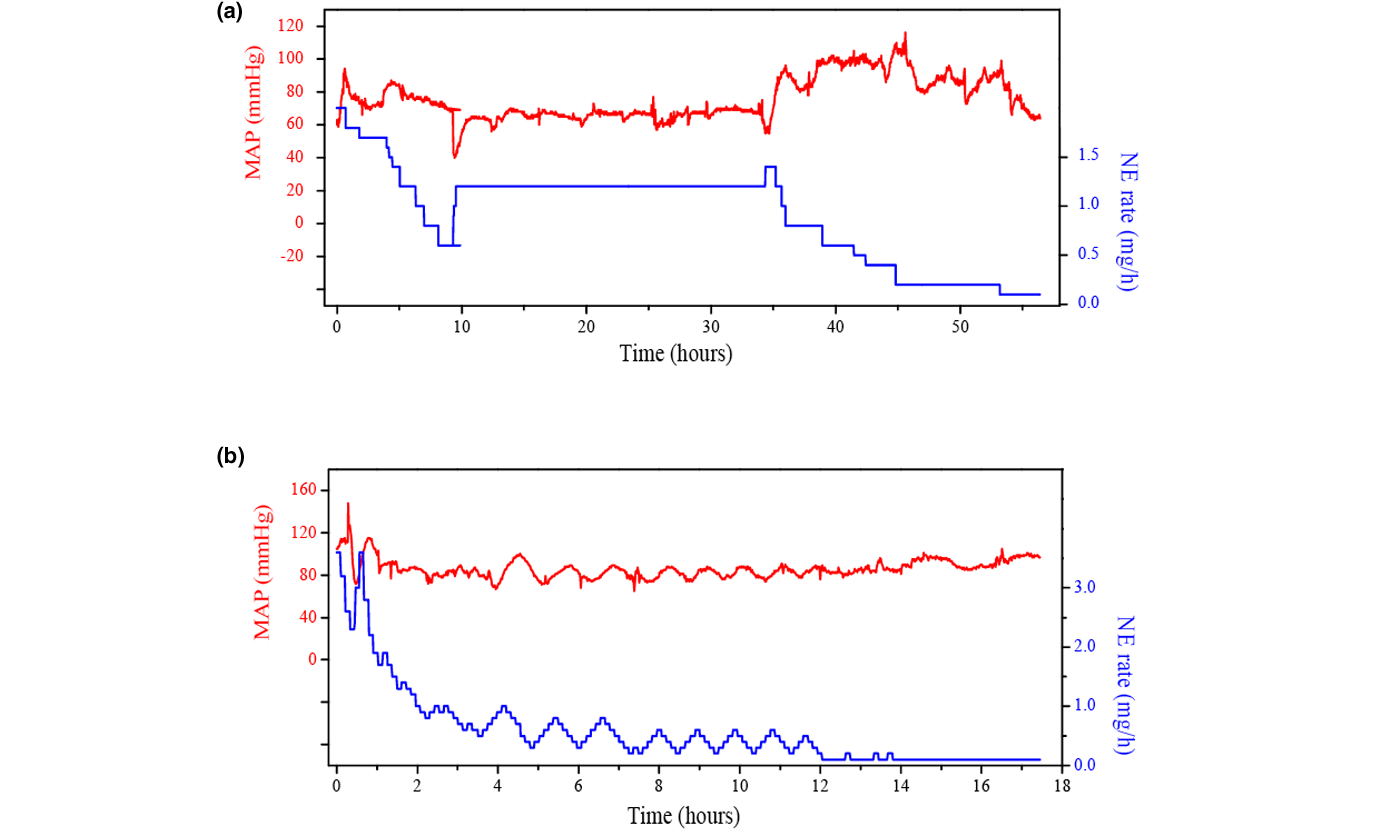
Time dependence in norepinephrine infusion rate and mean arterial pressure. **(a) **Norepinephrine (NE) infusion rate and mean arterial pressure (MAP) over time for a representative patient included in the control group. There is a linear decrease in norepinephrine infusion rate. **(b) **Norepinephrine infusion rate and MAP over time for a representative patient included in the fuzzy group. The change in norepinephrine infusion rate is more or less sinusoidal.

Figure 3 illustrates the changes in norepinephrine infusion rate for one patient from each group. Whereas there is a linear decrease in rate in the control group patient, the change is more or less sinusoidal in the fuzzy group patient.

## Discussion

Our study demonstrates a large and significant reduction in time of cessation of norepinephrine in patients enrolled in the group undergoing closed-loop control based on fuzzy logic. We attribute this reduction to closed-loop control of norepinephrine infusions. Moreover, the total amount of norepinephrine infused was significantly lower in the fuzzy group than in the control group. However, the reduced duration of shock was not associated with an expected reduction in mortality or in the duration of mechanical ventilation, perhaps because of the small numbers of patients included in the study.

Modern medicine is faced with the challenge of acquiring, analyzing and applying the large amount of knowledge necessary to solve complex clinical problems [14]. This pilot study supports the view that closed-loop control of norepinephrine administration is safe and feasible in intensive care. Currently, fuzzy logic, neural networks and genetic algorithms are three popular artificial intelligence techniques that are widely used in many medical applications [15]. Fuzzy logic is the science of reasoning, thinking and inference that recognizes and exploits real-world phenomenon that everything is a matter of degree. Instead of assuming that everything is black or white (conventional logic), fuzzy logic recognizes that in reality most things fall somewhere in between (that is, varying shades of grey) [14]. It was introduced by Lofti Zadeh in 1965 [16]. It uses continuous set membership from 0 to 1, in contrast to Boolean or conventional logic. Medicine is essentially a continuous domain, and most medical data are inherently imprecise. We chose a fuzzy logic algorithm because its successful use has been reported in many applications, for instance to control drug infusion to maintain adequate levels of anaesthesia, muscle relaxation, arterial pressure control, and patient monitoring and alarms [17]. In particular, fuzzy logic appears well suited to medical decision making in the ICU [18].

The main aim of septic shock treatment is to restore and maintain adequate tissue oxygenation [19]. This can only be achieved through appropriate control of the MAP and cardiac index. Norepinephrine is the vasopressor currently used to manage hypotension in patients with an optimal cardiac filling pressure. The first haemodynamic effect observed with norepinephrine is an increase in systemic vascular resistance and consequently in MAP through stimulation of α-receptors. Additional stimulation of β-receptors increases the cardiac index [20]. Vasopressors such as norepinephrine should be used only to restore resistance and/or MAP values to normal in patients with marked and documented vasodilatation [3].

The target MAP level is a value above 65 mmHg, which is perhaps a little higher than should be sought in patients with coronary risk factors [3,21]. We achieved and maintained this target with norepinephrine doses that we considered near optimal insofar as they were determined by a feedback system controlled by fuzzy logic. The total amount of norpinephrine administered was much reduced in the fuzzy group compared with the control group, probably because the physiological requirements for a drug with a very short half-life are better met by sinusoidal variation in infusion rate. This resulted in a lower total dose being administered and in an apparently shorter duration of septic shock.

Another advantage of fuzzy logic based closed-loop control of norepinephrine infusion was MAP during the weaning period. As shown in Figure 3, the patient's MAP slowly oscillates around the target value set by the intensivist in the fuzzy group; this is in contrast to the control patient, in whom it tends to drift, with more marked amplitudes. Nevertheless, the curve analysis did not reveal any statistically significant differences between the groups in terms of number of hypotensive episodes (defined as a MAP <55 mmHg; data not shown).

The rate of infusion rate modifications was empirically set at 7 minutes in order to take into account the equipment's inertia and patient's time to haemodynamic response. We estimated, during the study, the system's inertia by measuring the time separating a norepinephrine rate infusion peak from a MAP peak (see Figure 3). By using this method, we arrived at an estimate of 15 minutes. We therefore believe that this rate should be employed in further studies.

The shorter duration of shock may be due to a much reduced vascular response to α-agonists during the early phase of septic shock [22]. Studies *in vitro *and *in vivo *have suggested that α-adrenergic receptors (α1A, α1B and α1D) are downregulated at the level of gene expression and that this effect is mediated by proinflammatory cytokines [23]. Catecholamines, and in particular norepinephrine, also downregulate α-adrenergic receptors [24]. Prolonged exposure of human embryonic kidney cells to norepinephrine decreases the level of α1A-adrenergic receptor subunits at 48 hours by nearly 40% and of α1D-receptors at 24 hours by 51%. Similar results have been obtained in rabbit aortic smooth muscle cells. The decrease is observed after 4 hours of exposure and gives way to a gradual increase at 24 hours. It occurs in the wake of a decrease in the level of α1-adrenergic receptor mRNA [24]. The closed-loop control based on a fuzzy logic algorithm, by limiting exposure to norepinephrine, might preserve the function and pool of α1-receptor and thus lead to decreased patient resistance to norepinephrine infusion.

## Conclusion

We conclude that a closed-loop system based on fuzzy logic algorithm results in the use of much lower norepinephrine doses during weaning in patients with septic shock and leads to a decrease in the duration of weaning duration. By providing optimal delivery in relation to the individual patient's physiology, fuzzy control might constitute a better approach than constant flow infusion. Further studies in a larger number of patients are needed to confirm these results and to assess the effect of fuzzy control of norepinephrine infusion on morbidity and mortality.

## Key messages

• The weaning rate of catecholamines is usually chosen empirically by intensivists.

• A closed-loop control system based on fuzzy logic for norepinephrine infusion was associated with reduction in the duration of norepinephrine weaning in patients with septic shock.

• Fuzzy logic algorithm is a valid method to pilot an automated syringe pump in intensive care.

• The total amount of norepinephrine infused in the fuzzy group was significantly lower than that with manual control (the control group).

• Further studies are needed assess the effect of fuzzy control of norepinephrine infusion on morbidity and mortality in critically ill patients.

## Abbreviations

ICU: intensive care unit; MAP: mean arterial pressure.

## Competing interests

Frédéric Adnet received grant support from Boehringer Ingelheim and Sanofi-Aventis. The other authors declare that they have no competing interests.

## Authors' contributions

MM, BG and FA designed the study, analyzed and interpreted the data, and drafted the manuscript. FV, SWB, PK, JPF, C, FC and FL were responsible for data acquisition, analysis and interpretation of data. EV and CMG were responsible for data management and statistical analysis.

## Supplementary Material

Additional file 1 A pdf document that explains fuzzy logic theory. Click here for file
