# Supplementary material for: Norepinephrine weaning in septic shock patients by closed loop control based on fuzzy logic
Source: Crit Care. 2008 Dec 9;12(6):R155. doi: 10.1186/cc7149 (PMC2646320; doi:10.1186/cc7149)
Supplement: Additional file 1 — A pdf document that explains fuzzy logic theory. [file cc7149-S1.pdf]

Appendix

FUZZY SET THEORY

Fuzzy sets are sets whose elements are only partial members of the set. Fuzzy logic, introduced by Zadeh in the 1960s, is an extension of conventional logic that models the uncertainties of natural language, handling the concept of partial truth that is the true value between “completely true” and “completely false”. In traditional set theory, a value of a variable either belongs to a set or it does not, so the membership degree of a variable to a set can be only one or zero. Fuzzy set theory permits gradual membership of elements to a set from zero to one. The zero value represents a complete non-membership, the one value represents a complete membership, and values in between represent intermediate degrees of membership (Fig. a). The shape of the function can be triangular, trapezoidal, Gaussian, or an arbitrary curve (Fig. b). The function represents the fuzzy number, i.e. the degree of membership of the element.

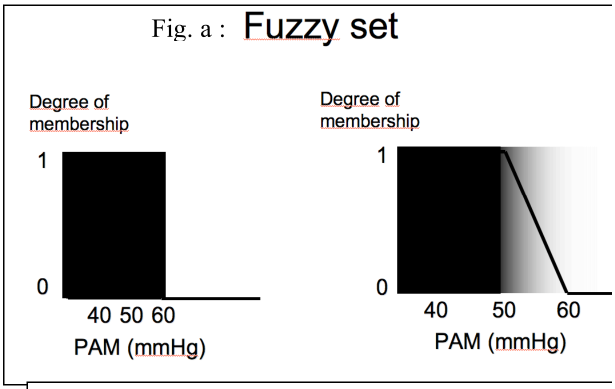

Fig. a : Fuzzy set

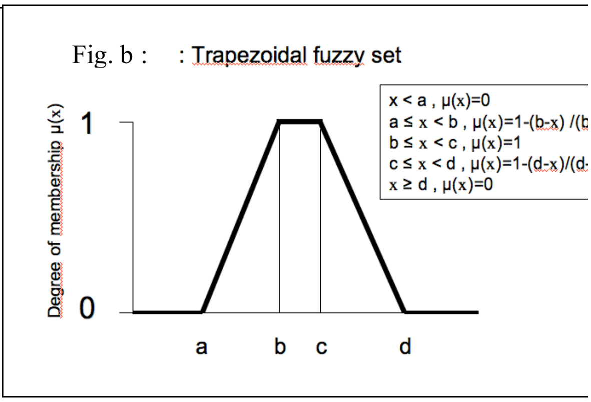

Fig. b : : Trapezoidal fuzzy set

Application of fuzzy set theory to control of mean arterial pressure

When applying fuzzy theory and fuzzy sets to mean arterial pressure and norepinephrine infusion, language terms such as normal, moderate hypotensive episode, increase norepinephrine dose, etc are transcribed into mathematical language (“fuzzification”). Fuzzy control indicates which actions have to be taken under a given set of outputs. Our output variables were mean arterial pressure (MAP) and variations in MAP (ΔMAP). The input variable was the adjustment needed in norepinephrine dose.

Step 1 : conversion of MAP and dosage into trapezoidal fuzzy sets.

For convenience and to simplify computation, we used trapezoidal fuzzy sets (Fig. b). Sets are denoted {a,b,c,d}.
Fuzzification of linguistic variables: MAP, ΔMAP and adjustment needed in norepinephrine infusion

Severe Hypotension: fuzzy set {ø,0,40,60}
Moderate hypotension: fuzzy set {50,60,60,70}
Normotension: fuzzy set {60,70,70,80}
Moderate hypertension: fuzzy set {70,80,80,100}
Severe hypertension: fuzzy set {80,100,200, ø }

Severe decrease in arterial pressure: fuzzy set { ø,-30,-20,-10}
Moderate decrease : fuzzy set {-20,-10,-10,0}
Stability : fuzzy set {-5, 0, 0,+5}
Moderate increase : fuzzy set {0,+10,+10,+20}
Severe increase : fuzzy set {+10,+10,+30, ø }

The adjustment needed in norepinephrine dose was similarly fuzzified (Fig. c).

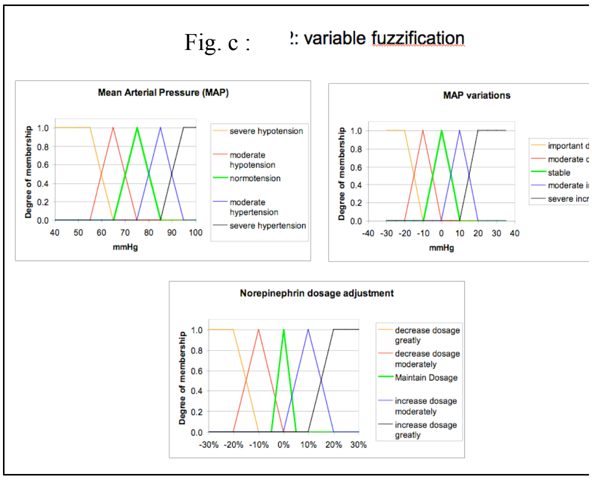

Fig. c : : variable fuzzification

Step 2: Defuzzification :

Defuzzification is the process of producing a quantifiable result in fuzzy logic. Rules on adjusting dosage might result in "Decrease infusion", “Maintain infusion”, “Increase infusion". Defuzzification transforms this result into a single number indicating the change in dose required. The centroid for the trapezoid was used as the defuzzification method according to an inference table. The *x* coordinate of the centroid is the defuzzified value. The program for acquisition, signal treatment and fuzzy control is written in visual basic 5 (Microsoft Corp©, Redmond, WA).
